# The management of acute venous thromboembolism in clinical practice – study rationale and protocol of the European PREFER in VTE Registry

**DOI:** 10.1186/s12959-015-0071-z

**Published:** 2015-10-21

**Authors:** Giancarlo Agnelli, Anselm K. Gitt, Rupert Bauersachs, Eva-Maria Fronk, Petra Laeis, Patrick Mismetti, Manuel Monreal, Stefan N. Willich, Wolf-Peter Wolf, Alexander T. Cohen, On behalf of the PREFER in VTE investigators

**Affiliations:** University of Perugia, Santa Maria della Misericordia Hospital, Perugia, Italy; Herzzentrum Ludwigshafen Med. Klinik B, Ludwigshafen, Germany; Klinikum Darmstadt GmbH, Darmstadt, Center of Thrombosis and Hemostasis, University of Mainz, Mainz, Germany; Center of Thrombosis and Haemostasis, University of Mainz, Mainz, Germany; Daiichi Sankyo Europe GmbH, Munich, Germany; CHU Saint-Etienne Hopital Nord, Saint Etienne, Cedex 2 France; Hospital Universitari Germans Trias i Pujol, Barcelona, Spain; Charité – Universitätsmedizin Berlin, Berlin, Germany; King’s College, Guys and St Thomas’ Hospitals NHS Foundation Trust, Westminster Bridge Road, London, SE1 7EH UK

**Keywords:** Venous Thromboembolism, Anticoagulation, Vitamin K antagonists, Novel Oral Anticoagulants, Prevention, Registry

## Abstract

**Background:**

Venous thromboembolism (VTE) is a major health problem, with over one million events every year in Europe. However, there is a paucity of data on the current management in real life, including factors influencing treatment pathways, patient satisfaction, quality of life (QoL), and utilization of health care resources and the corresponding costs. The PREFER in VTE registry has been designed to address this and to understand medical care and needs as well as potential gaps for improvement.

**Methods/design:**

The PREFER in VTE registry was a prospective, observational, multicenter study conducted in seven European countries including Austria, France Germany, Italy, Spain, Switzerland, and the UK to assess the characteristics and the management of patients with VTE, the use of health care resources, and to provide data to estimate the costs for 12 months treatment following a first-time and/or recurrent VTE diagnosed in hospitals or specialized or primary care centers. In addition, existing anticoagulant treatment patterns, patient pathways, clinical outcomes, treatment satisfaction, and health related QoL were documented. The centers were chosen to reflect the care environment in which patients with VTE are managed in each of the participating countries. Patients were eligible to be enrolled into the registry if they were at least 18 years old, had a symptomatic, objectively confirmed first time or recurrent acute VTE defined as either distal or proximal deep vein thrombosis, pulmonary embolism or both. After the baseline visit at the time of the acute VTE event, further follow-up documentations occurred at 1, 3, 6 and 12 months. Follow-up data was collected by either routinely scheduled visits or by telephone calls.

**Results:**

Overall, 381 centers participated, which enrolled 3,545 patients during an observational period of 1 year.

**Conclusion:**

The PREFER in VTE registry will provide valuable insights into the characteristics of patients with VTE and their acute and mid-term management, as well as into drug utilization and the use of health care resources in acute first-time and/or recurrent VTE across Europe in clinical practice.

**Trial registration:**

Registered in DRKS register, ID number: DRKS00004795

## Background

Acute venous thromboembolism (VTE), including deep-vein thrombosis (DVT) and pulmonary embolism (PE) is a common disorder with an annual incidence of approximately 1 or 2 cases per 1000 persons in the general population [1–3]. Patients with DVT and PE have increased morbidity and mortality both related to these conditions and also associated co-morbidities such as cancer, medical conditions and surgical procedures [4]. The main objective of anticoagulant therapy for patients with acute VTE is to prevent thrombus extension, embolization and recurrences. According to current practice guidelines the management of patients with acute VTE consists of an initial treatment with bodyweight-adjusted subcutaneous low molecular weight heparin (LMWH); adjusted-dose intravenous or fixed dose subcutaneous unfractionated heparin (UFH); or bodyweight-adjusted subcutaneous fondaparinux followed by long-term treatment with a vitamin K antagonist (VKA) or non-VKA oral anticoagulants (NOACs) [5]. For the treatment of PE the current 2014 European Society of Cardiology Guidelines on the diagnosis and management of acute PE recommend the use of NOACs as alternatives to VKAs [6].

Patients should receive parenteral anticoagulants (either LMWH or UFH or fondaparinux) for at least five days. It is recommended to start VKA on the first treatment day because of the slow onset of action. LMWH, UFH, or fondaparinux therapy may be discontinued when the VKA has reached its therapeutic level as indicated by an international normalized ratio (INR) ≥2 at two or more measurements at least 24 h apart. VKA therapy should be continued for at least 3 months. For most patients with a DVT and/or PE secondary to a transient risk factor the currently recommended duration of treatment is sufficient, although extension by another 3 to 6 months of therapy may be indicated in some patients [3]. However, for those with unprovoked DVT or PE, the recommendation is to evaluate the risks and benefits for prolonged therapy. In either case, the VKA dosage regimen needs to be adjusted to maintain the INR in the therapeutic range (target 2.5, range 2.0 to 3.0).

VKAs (such as the coumarins: warfarin, acenocoumarol or phenprocoumon) are indirect coagulation inhibitors, which act by blocking the vitamin K-dependent liver synthesis of the plasma coagulation factors II, VII, IX and X. They were the only oral anticoagulants available for over 50 years. Randomized controlled trials have shown that warfarin, the most commonly used VKA, targeted to an INR between 2.0 and 3.0, reduces the risk of recurrent venous thromboembolic complications in subjects with DVT or PE by 80% to 90% [5,7–9]. However, the use of VKAs is complicated by several inherent problems including a delayed onset of antithrombotic action; a narrow therapeutic window that requires close laboratory monitoring using the INR; an unpredictable and variable pharmacological response; and food and drug interactions requiring frequent monitoring and dosage adjustment [10].

Recently developed oral anticoagulants that are directed against factor Xa or thrombin (factor IIa) overcome some limitations of standard therapy including the need for injections of parenteral anticoagulants and for regular dose adjustments on the basis of laboratory monitoring [11–13]. However, VKAs are still often prescribed and although NOACs are widely approved in Europe, use of NOACs is limited by national guidelines and reimbursement. In Europe, little is known about which factors influence the individual VTE patients’ utilization of health care resources and the corresponding costs derived in hospitals, out of hospitals or in specialized centres during the period between confirmed first-time or recurrent VTE treatment and 12 months of follow-up.

### Study aims

The key aims of the PREFER in VTE registry were to assess the real-life acute and mid-term management of patients with VTE, the use of health care resources, and to provide data to estimate the costs for 12-months treatment following a first-time and/or recurrent VTE diagnosis in hospitals or specialized centers in Europe.

In addition, existing anticoagulant treatment patterns, patient pathways, clinical outcomes, treatment satisfaction, and health related quality of life (HR-QoL) were documented.

### Primary objectives

The primary objective of this registry was to assess the 12-months direct healthcare resource use and to provide data to estimate the costs following an acute first time or recurrent VTE. In addition, detailed insights into the patients’ characteristics, the management of acute VTE (in particular DVT and/or PE) with specific focus on prevention of VTE recurrences and its treatment-related events such as bleeding, recurrence of DVT/PE, myocardial infarction, stroke, systemic embolic events, post thrombotic syndrome, cardiovascular (CV) events, and death were collected. DVT was defined as DVT alone and PE as PE with or without DVT.

### Secondary objectives

The secondary objectives of this study were to 1) describe the treatment satisfaction, HR-QoL, and clinical outcomes following first time or recurrent VTE; 2) to explore the potential relationships between different anticoagulants and duration of therapy, resource use, estimated costs, treatment satisfaction, HR-QoL, and clinical outcomes; 3) to explore possible geographic variations in the management of VTE patients, duration of therapy, resource use, estimated costs and treatment satisfaction.

## Methods and design

**Fig. 1:**
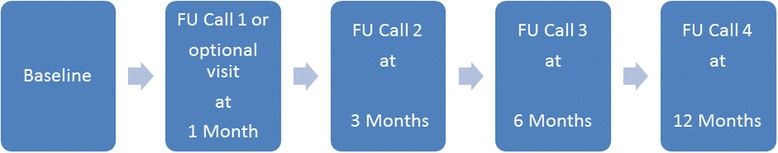
Time course of the data collection from baseline to 12 months follow-up

The PREFER in VTE registry was a prospective, observational, multicenter study with a follow-up of 12 months and enrolled 3,545 consecutive patients who gave informed consent from 381 centers (311 active centers enrolling ≥1 patient) in seven European countries including Austria, France, Germany, Italy, Spain, Switzerland, and the UK between January 2013 and July 2014. Data were recorded at baseline of the acute VTE and prospectively documented during follow-up at 1, 3, 6 and 12 months (Fig. 1).

Patient information was collected from two different sources: collection of baseline data took place in the hospitals or specialized centers at the time of the diagnosis of the acute VTE. Hospitals or specialized/primary care centers could also opt to follow-up the patients at 1 month. As hospital based investigators do not always see the patient during the following 12 months as part of the routine clinical care, patients were followed by telephone calls. Patients agreed to provide contact details for the planned follow-up calls with the permission to hand the contact details over to the respective local contact research organization (CRO).

This registry was conducted in accordance with the Declaration of Helsinki and adheres to the principles of Good Epidemiology Practice, and applicable regulatory requirements. The responsible ethics committees of the participating countries and the hospital-based institutional review boards approved the protocol of this registry. Patients enrolled into this registry provided written informed consent. Due to the nature of a non-interventional registry, no specific treatments, tests, or procedures were mandated or withheld from the patients. Treatment pattern and treatment initiation, continuation or changes were solely at the discretion of the physician and the patient. There was no attempt to influence the prescribing patterns of any individual treating physician. All medication was prescribed in the usual standard of care and was not provided by the study sponsor. Participation in the study in no way influenced payment or reimbursement for any treatment received by patients during the study. Patients were free to withdraw from the registry at any time.

### Selection of sites

Investigator sites were representative for the specific local distribution of primary and secondary care settings in each participating country. A sufficient number of sites were identified including hospitals and specialized centers to best represent current practice of VTE diagnosis and treatment in each particular country. Most centers contacted for participation were randomly selected, others were chosen from experienced centers and all centers were asked to provide institutional details in a short site feasibility questionnaire before being selected. Each active site consecutively recruited at least 1 and up to 60 patients. The enrollment period was 12 months.

### Selection of patients

Patients from Austria, France, Germany, Italy, Spain, Switzerland and the UK who gave written informed consent were consecutively enrolled into the registry in hospitals or specialized centers if they were at least 18 years old, had a confirmed first time or recurrent symptomatic VTE defined as either distal or proximal DVT, PE [3] or both, provided telephone contact details for follow-up calls, and were not simultaneously participating in a double blind interventional study.

We expect the parameters measured will vary between patients on the conventional pathway of treatment and those using NOACs and therefore the comparison between those following the conventional pathway and those on the NOACs and the impact of NOACs on QoL, Resource consumption and mostly health care utilization will be examined.

**Fig. 2:**
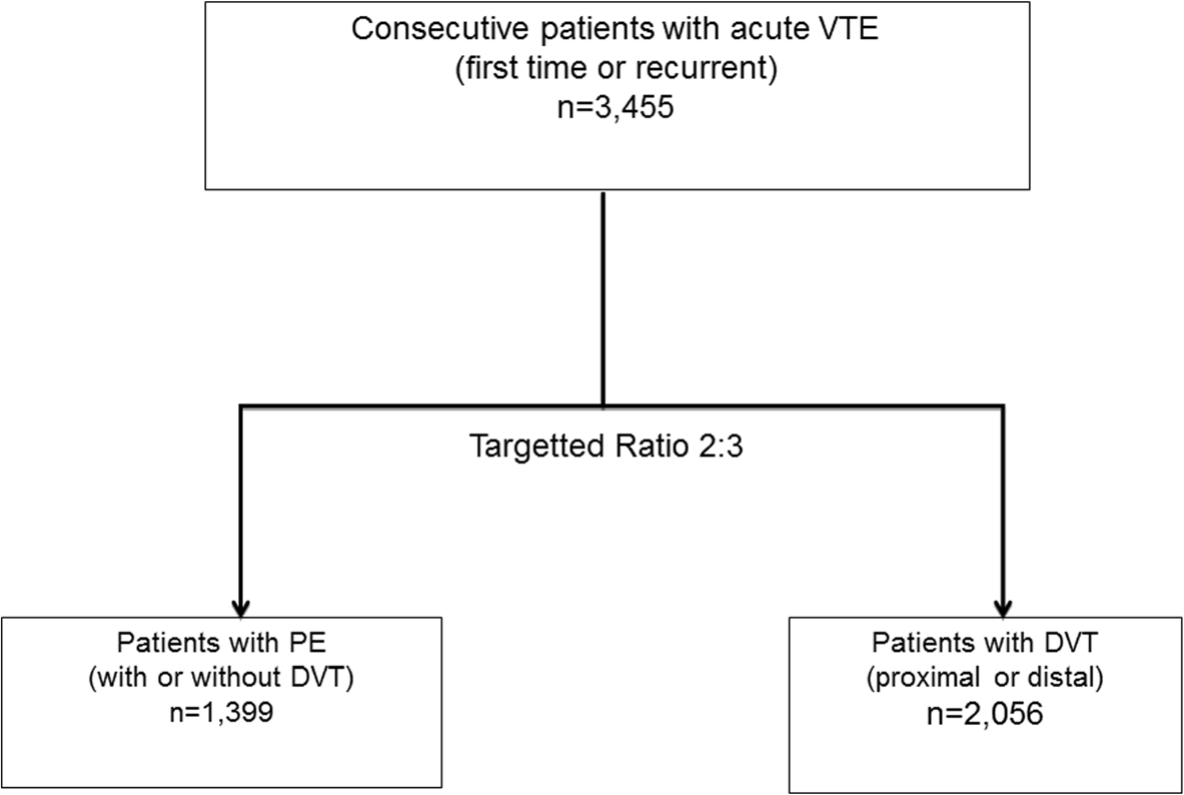
Patient enrollment

The study aimed to a achieve a general ratio of PE:DVT which was approximately 2:3 resulting in a total number of PE (with or without DVT) patients of *n* = 1,399 and a total number of patients with DVT (distal or proximal) of *n* = 2,056 (Fig. 2).

### Documented variables

The enrolling study site documented all patient related baseline data at the time of the acute VTE. The follow-up calls were performed centrally by local CROs. The CROs used standardized questionnaires to get all required information (i.e. recurrence of VTE, bleeding, post-thrombotic syndrome, death, hospitalization, as well as medications and treatments, QoL and patient satisfaction (Perception of Anticoagulant Treatment Questionnaire 2 [PACT-Q2], Venous Insufficiency Epidemiological and Economic Study [Veines-QoL/Sym] or Pulmonary Embolism Quality of Life [PEmb-QoL]), resource consumption / health care utilization). Patient diaries were distributed to facilitate the standardized structured phone calls. The treatment satisfaction questionnaire (PACT-Q2), the disease specific questionnaire (PEmb-QoL or Veines-QoL/Sym) and the QoL questionnaire (EQ 5D–5L) were filled out at baseline, and repeated by post at 1, 3, 6 and 12 months after first diagnosis of acute VTE.

Table 1 displays the variables documented at baseline and at the follow-up visits at 1, 3, 6 and 12 months. The data sources used in this study were clinical records, self-reports, claims, and data from telephone interviews.

**Table 1:** Data documented at baseline and at 1, 3, 6 and 12 months follow-up

Variable	Baseline	Follow Up			
		Month 1	Month 3	Month 6	Month 12
Eligibility criteria ^(1)^	X				
Baseline information ^(2)^	X				
VTE risk factors and co- morbidities ^(3)^	X				
Baseline information on VTE ^(4)^	X				
VTE therapy ^(5)^	X	X	X	X	X
Current and previous treatment for prevention of stroke and other thromboembolic events ^(6)^	X	X	X	X	X
Quality of life ^(7)^	X	X	X	X	X
Patient satisfaction ^(8)^	X	X	X	X	X
PEmb-QoL Questionnaire (only PE-patients)	X	X	X	X	X
Veines-QoL/Sym Questionnaire (only DVT-patients)	X	X	X	X	X
Resource utilization ^(9)^	X	X	X	X	X
Clinical events and hospitalizations ^(10)^	X	X	X	X	X
Number of days in hospital, work days lost due to VTE, need for nursing/informal help	X	X	X	X	X

Legend

(1) Objectively confirmed first-time or recurrent VTE; age ≥ 18 years, written informed consent

(2) (Socio-) demographic variables: age, gender, height, weight, BMI, blood pressure, graduation, education, employment status, insurance status

(3) Major surgery, bleeding history medical illnesses, multiple trauma, hip fracture, lower extremity paralysis, previous VTE, increasing age, cardiovascular or respiratory failure, prolonged immobility, presence of central venous illness, estrogens, wide Varity of inherited and acquired hematological conditions, cancer, chemotherapy etc

(4) Date of first diagnosis, lead symptoms, diagnostic pathways, severity

(5) Thrombolysis, Heparin, Vitamin K Antagonist, Embolectomy, Catheter, Insertion of V.cava filter

(6) Physician’s clinical impression of the risk of stroke/ thromboembolic events; physician’s use of algorithm to determine risk; current anticoagulation (by drug, with information on continuation after the visit); discontinued anticoagulation (last 12 months); (Relative) contraindications to anticoagulation; INR (target and achieved value, frequency of tests, percentage of values within target range) D

(7) EQ-5D

(8) PACT-Q2

(9) Number of physician contacts (own office, other physicians); VTE related productivity loss and costs; number and type of VTE diagnostic tests since last visit /call

(10) Specifically due to: VTE, stroke, acute coronary syndrome including myocardial infarction, arterial embolism, decompensated heart failure, syncope, bleeding events

### Patients’ treatment satisfaction and quality of life

Data on patients’ treatment satisfaction and QoL were documented using specific patient-questionnaires. The following questionnaires were part of the evaluation:

#### Quality of Life Questionnaire (EQ 5D–5L)

EQ 5D-5L is a standardized measure of health status developed by the EuroQol Group in order to provide a simple, generic measure of health for clinical and economic assessment [14]. Applicable to a wide range of health conditions and treatments, it provides a simple descriptive profile and a single index value for health status that can be used in the clinical and economic evaluation of health care as well as in population health surveys.

#### Treatment Satisfaction Questionnaire

Anticoagulation-specific questionnaire PACT-Q. The ‘Perception of AntiCoagulant Treatment Questionnaire’ was developed to assess patients’ expectations of, and satisfaction with their anticoagulant treatment [15–17].

#### Veines-QoL/Sym

The Veines-QoL/Sym is a patient-based questionnaire designed for self-completion and measures the impact of DVT on symptoms and QoL from the patient’s perspective; this was only completed by patients with DVT [18].

#### PEmb-QoL

The PEmb-QoL questionnaire was modeled on the QoL after DVT (Veines-QoL/Sym) questionnaire. This questionnaire, like the Veines-QoL/Sym, assesses the frequency of symptoms, the time of day which the complaints are their worst, and activities of daily living, as well as work-related problems. However, the PEmb-QoL questionnaire is distinct from the Veines-QoL/Sym in the inclusion of pulmonary-specific symptoms, adding questions on limitations in daily physical activities, and extending the number of questions on emotional functioning [19].

The PEmb-QoL was only filled out by patients with PE.

### Data management and quality control

Data entry was performed by the physician or study nurse via a secure website directly into an electronic database. This approach allowed online checks for plausibility and integrity.

There were three strategies for data quality checks: validations that occurred at the time of data entry (i.e., “front-end”), a second, more sophisticated quality control program that ran as a prelude to the creation of the analysis data set and on-site data monitoring by the CRO.

Front-end data checks are advantageous because mistakes are caught and corrected at the time of entry. Certain data elements can be required, while other elements may allow missing values. Additionally, parameters were defined to allow entry of only those records that met the inclusion criteria.

Thirdly, prior to the creation of the analytic dataset, more extensive quality control processes were performed. These checks, programmed in SAS (release 9.2 or higher; Cary, NC, USA), included parent–child edits, consistency edits, and data transformations that facilitated analyses.

### Sample size

In order to provide meaningful data on reimbursement from different agencies in each participating country, and because significant variation was expected between countries, special care was taken to determine a suitable sample size of patients for each country. As costs are difficult to determine directly and are mostly triggered by the hospitalization of the patients, the sample size calculation was based on the rate of adverse events (AEs) leading to, or prolonging, hospitalization.

Assuming a rate for the AEs that are leading to or prolonging hospitalization and an absolute precision Δ, the sample size N is calculated which is needed if the respective 95% confidence interval (CI) should have a length/precision of 2Δ (±Δ). Concretely, for DVT a hospitalization rate of 12.3% was assumed [15], while for PE a hospitalization rate of 17.9% was taken [16]. Furthermore, for DVT as well as for PE, a relative precision of 25% was assumed which corresponded to an absolute precision of 0.031 for DVT and an absolute precision for PE of 0.045. Based on these assumptions a minimum of 432 evaluable DVT patients were needed while for PE a minimum of 279 evaluable patients were needed (Table 2). Taking into account a dropout rate of 20% and being aware of the fact that DVT and PE patients were recruited in a ratio of 3:2 (DVT: PE), this resulted in a total of 900 patients recruited (= 540 DVT patients + 360 PE patients) in each region and 4,500 patients in the overall registry. The above sample size consideration is taken from the observational plan and was made before the start of the study. The primary parameters of interest were the “costs” and the “rate for AEs leading to or prolonging hospitalization” was taken as the main trigger for this very general parameter. Therefore, the sample size consideration should be taken as a general guidance and needs to be interpreted in a flexible way. Based on real-life experience, in general a sample of ~600 patients is considered as robust enough for reliable cost estimations.

**Table 2:** Sample size calculations

	Assumed rate of AE leading to, or prolonging, hospitalization	Assumed absolute precision (relative precision)	Sample size (without drop-outs)	Sample size (including drop-outs)
DVT	0.123	0.031 (± 25%)	432	540
PE	0.179	0.045 (± 25%)	279	349

Sample size (for each region) for the assumed rate of AEs leading to, or prolonging hospitalization 0.123 (DVT) and 0.179 (PE) in order to achieve an absolute precision of 0.031 (DVT) and 0.045 (PE) for a two-sided 95% CI. (Based on normal approximation, calculated by nQuery Advisor^®^ 7.0.). AE, adverse event

### Statistical analysis

This registry collected data under real life conditions. The statistical analysis was performed in an explorative and descriptive way. All variables collected in the electronic case report form as well as the data obtained from the QoL assessments and all derived parameters were used in the statistical analysis.

Binary, categorical, and ordinal parameters were summarized by means of absolute and percentage numbers within the various categories.

Numerical data was summarized by means of standard statistics (i.e. number of available data, number of missing data, mean, standard deviation, minimum, median, maximum, lower and upper quartile).

Time-to-event variables were analyzed via a Cox proportional hazard regression model presenting hazard ratios and the corresponding 95% CIs. No formal statistical tests were performed within the statistical analysis.

A biometrical report using descriptive statistics of all documented parameters was used to describe the overall patient population as well as for each participating country or by region. Patients violating any inclusion/exclusion criteria were identified and documented. There were several variables of interest for additional analyses including: gender, age, weight, diabetes. Details on the selection criteria used were given in the statistical analysis plan and in the statistical section of the report.

As the registry was exploratory in nature, and no hypothesis testing was conducted, there were no significance levels to be adjusted, and there was no requirement to provide stopping rules for the study.

The statistical analysis was performed using SAS (release 9.2 or higher; Cary, NC, USA).

## Discussion

Little is known about populations of unselected patients with VTE. In Europe there is only minimal data describing individual patient characteristics, management patterns, factors influencing health care resource utilization and the corresponding costs. For completeness these data need to be derived from confirmed cases within hospitals, out of hospitals, in primary care or in specialized centers. The critical period for recurrences and bleeding is the time between first diagnosis and the following 12 months. Including unselected patients with both first time and recurrent VTE allows the burden of disease to be quantified.

The PREFER in VTE registry will help to get a detailed insight into the characteristics and management of patients with VTE (DVT and/or PE) with focus on prevention of events (bleeding, recurrence of DVT, recurrence of PE, post thrombotic syndrome, CV events, other complications and death) in a real life setting. Data were collected from VTE patients during the acute event and followed by an observational period of up to 1 year and were used to assess direct healthcare resource use and estimated costs following acute first-time or recurrent VTE. Patients were therefore treated on the discretion of the physician in charge according to their regular medical care.

In the past, most of the published information on the current therapy and natural history of patients with VTE comes from randomized clinical trials, with strict inclusion and exclusion criteria, fixed doses of anticoagulant drugs, and limited follow-up, mostly focused to obtain data on efficacy and safety. However, a number of patients with VTE are never recruited in clinical trials, particularly, the very young, the very elderly, the pregnant, those with multiple comorbidities, those with a high risk of bleeding and those with polypharmacy. Also there is scarce information on the current therapy in real life (drugs, doses, duration and approach to patients with VTE recurrences and/or bleeding complications). There is also limited information regarding the QoL, utilization of resources, costs, satisfaction of the patient and influence of their willing on the duration of therapy. This information may be very useful for patients, clinicians, healthcare providers, health technology assessors and to the pharmaceutical industry, who will all benefit from the data about many aspects of VTE not previously considered in detail in a broad unselected population. Currently, PREFER in VTE is the only registry evaluating the management of acute VTE in Europe; on an international level, the management of acute VTE has been assessed in the RIETE registry and in the ongoing GARFIELD-VTE registry [20,21].

The observational PREFER in VTE registry aims to address these questions by describing the current anticoagulant treatment patterns, pathways of clinical care, clinical outcomes as well as patient’s treatment satisfaction and HR-QoL in a real world setting. Real life data are of utmost importance to collect information on the safety, efficacy and drug utilization of any novel drug and to link the data with the regulatory mandated phase III data. This is an important stage in the observation and stringent control of all novel drugs. Therefore, there is a need for a European Union wide, international observational registry of VTE patients treated in everyday clinical practice to provide complementary data to that from the trials and to fully understand the treatment pathway of patients.

This large, European registry of acute VTE patients allows the opportunity to answer several research questions that have not previously been investigated within a non- randomized, non-selected population. These questions will address the following six areas:

1. Patient characteristics of consecutive patients with acute VTE in clinical practice including comorbidities, history of thromboembolic/bleeding events, temporary and permanent risk factors including severe medical disorders and interventions with VTE risk
2. Diagnosis of acute VTE (first-time [initial] or recurrent) including timing, symptoms and signs, clinical status and severity
3. Patient pathways including referral details and related time intervals (from primary to secondary care, e.g., internist, hematologist, vascular surgeon or physician, cardiologist and/or hospital) as well as diagnostic pathways
4. Drug utilization/use pattern of drugs for treatment of VTE and prevention of related events
5. Health related quality of life including patient satisfaction and real-world assessments of quality of life
6. Resource consumption/Health care utilization.

## Conclusions

The PREFER in VTE registry will provide valuable insights into all these factors in patients with VTE and their acute and mid-term management. The study will provide the opportunity to identify differences in management and outcomes across care settings, and will offer clarity relating to the effectiveness of anticoagulation treatment strategies to treat acute VTE and to prevent recurrent VTE events.

## Appendix

List of PREFER in VTE investigators.

### Austria

Marianne Brodmann, Peter Rief, Universitätsklinikum Graz, Graz; Lisbeth Eischer, Slagjana Stoshikj, Medizinische Universität Wien, Wien; Michael Hirschl, Serge Weinmann, Landesklinikum Zwettl, Zwettl; Peter Marschang, Universitätsklinik Innsbruck für Innere Medizin, Innsbruck.

### France

Fabrice Abbadie, Centre hispitalier Jacques Lacarin, Vichy; Antoine Achkar, CHI Eure et Seine – Site de Vernon, Vernon; Azeddine Addala, Perrone Reynaldo, Lyon; Frédéric Adnet, Hopital Avicenne, Bobigny; Jean-François Alexandra, Hopital Bichat Claude Bernard, Paris; Sandro Aquilanti, Espace ARTOIS Santé, Arras; Abdelkader Belhassane, Centre Hospitalier de Cambrai, Cambrai Cedex; Anne Benaroya, Pole Sante Des Aspres, Thuir; Toufek Berremili, Marie Chevallier Grenot, CH Annecy Genevois, Pringy Cedex; Virginie Birr, Daniela Holtea, CHU Emile Muller, Mulhouse Cedex; Christophe Bonnin, Le Gibraltar 10–12, Nice; Frederic Bosler, 19 rue Robert Schumann, Sarrebourg; Marie-Gabrielle Bresin Durand, Résid des remparts 7 rue des lombards, Hennebont; Dominique Brisot, Centre Commercial La Croisee, Clapier; Christophe Brousse, Tarodo De La Fuente, Clinique Du Parc, Castelnau-le-Lez; Richard Cayman, 8 rue des des de l'aire, Bourgoin Jallieu; Michèle Cazaubon, 17, rue Mesnil, Paris; Olivier Champion, 6 Avenue Georges Pompidou, Chalon-sur-Saône; Myriam Chanut, Immeuble Le Clos de Bellande, Aubenas; Pascal Chevalet, Hopital Bellier, Nantes; Jerome Connault, Cecile Durant, CHU Hôtel-Dieu de Nantes, Nantes; Joel Constans, Hopital St Andre, Bordeaux; Mihaela Cordeanu, CHRU Strasbourg, Strasbourg; Francis Couturaud, Karine Lacut, CHRU Hopital Cavale Blanche, Brest; Laure De Dedker, François Xavier Piloquet, Hôpital Nord Laennec, Saint Herblain; Eric Decoulx, Centre Hospitalier de Tourcoing, Tourcoing; Benoit Derrien, Centre Hospitalier, Le Mans Cedex 9; Jean-Marc Diamand, Centre De Medecine Vasculaire, Grenoble; Antoine Diard, 25 Route De Creon, Langoiran; Youssef Douadi, Centre Hospitalier De Saint Quentin, St Quentin; Stephane Dupas, Santhi Samy Modeliar Remond, Marie-Antoinette Sevestre, CHU Amiens Picardie - Hôpital Sud, Amiens Cedex 1; Stéphane Edhery, Hôpital Saint Antoine, Paris; Nicolas Falvo CHU Dijon Service De Medecine Interne, Dijon; Claudia Farcas Taralunga, Hôpital Saint Charles, Toul; Emile Ferrari, Hôpital Pasteur, Nice; Catherine Gaillard, 14 rue Maurice Devillers, Peronne; Damien Garrigues, Clinique Monie, Villefranche-de-Lauragais; Jean Luc Gillet, 51BIS Avenue Professeur Tixier, Bourgoin Jallieux; Pascal Giordana, L'empire 29, Boulevard Dubouchage, Nice; Claire Grange, Denis Vital-Durand, Hopital Lyon Sud, Pierre Benite; Frédérick Grare, Espace Medical Torremila, Perpignan; Amine Hadj Henni, CHU Robert-Debré, Reims; Simone Heuser, Jeannot Schmidt, Centre Hospitalier Universitaire, Clermont Ferrand; Valérie Hidden-Henic, Centre Hospitalier de Wattrelos, Wattrelos; Delphine Hottin, Centre Hospitalier Docteur Schaffner, Lens Cedex; Bernard Imbert, Gilles Pernod, Hopital Nord Michallon, La Tronche; Daniel Jakob, Clinique Générale, Valence; Vincent Jacquinandi, Maison Médicale de Spécialistes, Trelaze; Christine Jurus, Clinique du Tonkin, Villeurbanne; Amelie Lacoste, 10, rue Abert Camus, Pontarlier; Jean-Pierre Laroche, Médipole, Avignon; Myriam Martin, 13 Rue De La Poste, Annecy; Cyrille Mazollier, Pôle Médical, Saint Laurent de la Salanque; Tahar Mersel, 2 rue Egassiairal, Narbonne; Gilles Miserey, 55 Rue Gambetta, Rambouillet; Charles Nedey, Clinique Du Tonkin, Villeurbanne; Monica Nou, Isabelle Quere, Hopital Saint Eloi, Montpellier; Pierre Ouvry, Clinique MEGIVAL, St Aubin sur Scie; Bernadette Peuch, 1 bis Place Mendes France, Castelnau le lez; Olivier Pichot, Centre de Médecine Vasculaire, Grenoble; Veyre Poulain, 19 rue Vimaine, Vienne; Patrick Ray, Hopital Thonon, Paris; Abed Rifai, Centre Hospitalier, Arras; Pierre-Marie Roy, Angers; Jean-Claude Saby, Clinique du Tondu, Bordeaux; Frédéric Simon, 51 avenue Jean JAURES, Lyon; Eliane Simonot-Lalandec, 60 bis rue de Camtimpré, Cambrai; Dominique Stephan, CHRU Strasbourg, Strasbourg; Anne Tissot, Clinique du TONKIN, Villeurbanne; Hubert Vodoungnon, Hôpital de Dunckerque, Dunkerque.

### Germany

Annette Adamczyk, Saskia Schnabl, Universitäts-Hautklinik Tübingen, Tübingen; Wail Al Ahmad, Heinz Weber, Sozialstiftung Bamberg, Bamberg; Christoph Axthelm, Praxis Axthelm, Pirna; Rupert Bauersachs, Klinikum Darmstadt, Darmstadt; Klaus Bergmann, Nägelsbachstraße 49 c, Erlangen; Ulrich Beschorner, Matthias Knittel, Herzzentrum Bad Krozingen, Bad Krozingen; Karl-Heinz Binias, Martin Pasligh, Ameos Klinikum Schönebeck, Schönebeck; Mehmet Boral, Friederike, Girke, Vivantes Klinikum Neukölln, Berlin; Hermann Bratsch, P7, 13, Mannheim; Gunter Brauer, Berliner Str. 158, Cottbus; Sebastian Burghard, Carl-von-Basedow-Klinikum, Merseburg; Christiane Demann, Carsten Rennebaum, Praxis Emter/Rennebaum/Demann, Hannover; Anita Demmig, Lindenallee 7, Hoppegarten; Ulrich Eberlein, Ketschengasse 22-24, Coburg; Frank Enger, MVZ Nibelungen (Enger), Biebesheim; Jens, Eschenburg, Jens-Uwe Eschenburg, Neubrandenburg; Lutz Forkmann, MVZ Polymed Forkmann, Chemnitz; Jürgen Frank, In der Schaf 14, Baesweiler; Holger Freischmidt, Martin Gassauer, BGU Ludwigshafen, Ludwigshafen; Ilona Fritsche, Cornelia Kubicek–Hofmann, Vivantes Klinikum im Friedrichshain, Berlin; Mark-Claudius Goebels, Marien-Hospital Euskirchen, Euskirchen; Stephan Guggenbichler, Stephan Guggenbichler, Frauenstrasse 17, München; Dirk Härtel, Klinikum Detmold, Detmold; Karsten Hartmann, Venenzentrum Freiburg, Freiburg; Peter, Heilberger, Heilberger, Schweinauer Hauptstr. 12, Nürnberg; Andreas Heinsius, Jüdisches Krankenhaus Berlin, Berlin; Michael Held, Steffen Schnupp, Klinikum Coburg, Coburg; Georg Herman, Am Finkenhügel 1, Osnabrück; Jörg Herold, Universitätsklinikum Magdeburg, Magdeburg; Frank Hertrich, Am Bahnhof 6, Lebach; Henrike Hommel, Privatklinik Robert Schindlbeck, Herrsching; Guntram Hütte, Christoph Kalka, Marienhospital Brühl, Brühl; Katja Jungandreas, Mathias Ramthor, Vivantes Humboldt Klinikum, Berlin; Jan Karcher, Nicolas Werner, Klinikum Ludwigshafen, Ludwigshafen; Susanne Karl-Wollweber, St. Marien-Hospital Lünen, Lünen; Dag-Alexander Keilhau, Lerchenfeld 14, Hamburg; Kerstin Kittel, Naumburger Str. 74, Weißenfels; Tanja Knolinski, Bethesda Krankenhaus Bergedorf, Hamburg; Christina Köhler, Sebastian Werth, Uniklinik Dresden, Dresden; Ute Kopplin, Kirchplatz 10a, Bad Salzungen; Ines Körner, Kathrin Wittig, Praxis Dres. Wittig/Rühlmann/Körner, Leipzig; Knut Kröger, Theodoros Moysidis, Helios Klinik Krefeld, Krefeld; Ulf Kroschel, Herzklinik Ulm, Ulm; Matthias Leschke, Tim zur Nieden, Klinikum Esslingen, Esslingen; Gerhard Lübbert, Krankenhaus Neu-Mariahilf GmbH, Göttingen; Arno Lutz, Petra Wucherpfennig, Vinzenzkrankenhaus Hannover, Hannover; Geert-H Marencke, Bremerhaven; Kai Mortensen, Michael Reppel, Universitätsklinikum Lübeck, Lübeck; Heike Nelles, Lindenaustr. 5, Altenburg; Kay Nestler, Kleiststr. 5, Grimma; Axel Neumeister, Alexander Schlosser, Helios Klinikum Erfurt, Erfurt; Wolfram Oettler, Carolusstr. 214, Görlitz; Ilka Ott, Deutsches Herzzentrum München, München; Annegret Otto, Alexander-Puschkin-Platz 4c, Riesa; Astrid Pertermann, Polimed Leipzig, Leipzig; Rahel Pfister, Kaiser-Joseph-Str. 262, Freiburg; Lukas Pindur, Gefäßpraxis Leverkusen – Linz, Leverkusen; Siamak Pourhassan, Chirurgische Gemeinschaftspraxis, Klosterstr. 12, Oberhausen; Diethard Predel, Grimmelallee 2c, Nordhausen; Thomas Pudollek, Wiesenstr. 1, Bernsdorf; Dietrich Reimer, Julius-Lübke-Str. 13, Anderbeck; Cornelia Richter, R 1, 2-3, Mannheim; Eberhad Rieker, Südstr. 29, Lauffen; Gabriele Rothenbücher, Zeppelinstraße 16, Dornstadt; Brigitte Rothhagen, Gothaer Str. 1, Waltershausen; Simone Rudolff, Markus Stücker, Maria-Hilf-KH Bochum (Venenzentrum), Bochum; Andreas Schäfer, Kristina Sonnenschein, Medizinische Hochschule Hannover, Hannover; Wolfgang Schafnitzl, Mönckebergstr. 18, Hamburg; Sebastian Schellong, Birgit Voigts, Krankenhaus Dresden-Friedrichstadt, Dresden; Martin Schiller, Klinikum Ingolstadt, Ingolstadt; Thomas Schmeink, Praxis Schmeink, Aachen; Henrik Schneider, Sana Hanse Klinikum Wismar, Wismar; Norbert Schön, Töginger Str. 27, Mühldorf; Matthias Schulze, Asklepios Klinikum Schwalmstadt, Schwalmstadt; Udo Sechtem, RBK Stuttgart, Stuttgart; Sabine Sedl, Feldstraße 23a, Schönberg; Harriet Simone Werno, Parcside medical center - Harriet Simone Werno, Nürnberg; Jörg Stachowitz, St. Johannisstift Paderborn, Paderborn; Marcus Thieme, Medinos Klinik Sonneberg, Sonneberg; Christiane Tiefenbacher, Marien-Hospital Wesel, Wesel; Dimitrios Tsantilas, Halderstr. 23, Augsburg; Petra Vieth, Marienhospital Steinfurt, Steinfurt; Jürgen vom Dahl, Katharina Grün-Himmelmann, Krankenhaus St. Franziskus Mönchengladbach, Mönchengladbach; Peter von Bilderling, Thordis von Maltik, Gefäßpraxis München – Mietaschk, München; Katrin Weinrich, Klinikum Augsburg, Augsburg; Marc Weyer, Eduardus Krankenhaus, Köln, Marc Weyer, DRK Kamillus Klinik, Asbach; Peter Wirtz, Kreiskrankenhaus Mechernich, Mechernich; Ina Wittig, Käthe-Kollwitz-Str. 9, Leipzig; Petra Zierock, Vivantes Klinikum Spandau, Berlin.

## Italy

Walter Ageno, Monica Caprioli, Elena Rancan, U.O. Medicina Interna I, Varese; Giancarlo Agnelli, Francesco Guercini, Valeria Mommi, Dipartimento di Medicina Interna e Vascolare, Perugia (PG); Maria Amitrano, Francesca Cannavacciuolo, Dipartimento Medicina Interna, Avellino; Maurizio Amore, Sebastiano D'Antoni, Ospedale Vittorio Emanuele II, Catania; Ermanno Angelini, Saverio La Forgia, Cardiologia-UTIC, Brindisi (BR); Pier Luigi Antignani, Giuseppe Calandra, Centro Vascolare, Roma; Andrea Arone, Francesco Perticone, Angela Sciacqua, Unità Operativa di Malattie Cardiovascolari Policlinico Mater Domini di Catanzaro, Catanzaro; Giovanni Asaro, Mario Bellisi, U.O. Chirurgia Vascolare, Palermo; Maria Teresa Attanzio, Antonio Pinto, U.O.C. Medicina Vascolare, Palermo; Valerio Attinasi, Enrico Cillari, Sabrina Sorvillo, P.O. Cervello, Palermo; Alberto Balbarini, Claudia Santini, Caterina Violo, U.O. Cardio- Angiologia Universitaria, Pisa; Elena Banfi, Corrado Lodigiani, Istituto di Ricovero e Cura a Carattere Scientifico –Istituto Clinico Humanitas, Rozzano (MI); Doris Barcellona, Stefano Delpin, Silvia Marongiu, Medicina interna 1 e emocoagulopati, Monserrato (Cagliari); Giovanni Barillari, Samantha Pasca, Azienda Ospedaliero Universitaria S. Maria della Misericordia, Udine; Claudia Bartolini, Paolo Verdecchia, Ospedale di Assisi, Assisi (PG); Mosè Bartone, Gerardo Mancuso, Unità Operativa Complessa di Medicina Interna - Presidio Ospedaliero “Giovanni Paolo II”, Lamezia Terme (CZ); Ignazio Bellanuova, Salvatore Felis, Ospedale Garibaldi Centro – Catania, CATANIA (Catania); Annamaria Bellizzi, Luca Masotti, AUSL 6 di Livorno, Cecina (LI); Marina Bianchi, Anna Carugati, Ospedale Valduce, Como; Giuseppe Bianchini, Giorgio Guarnera, Istituto Dermopatico dell’Immacolata, ROMA; Benedetta Boari, Massimo Gallerani, Mauro Pasin, Arcispedale Sant’Anna di Ferrara, Ferrara; Cristiano Bortoluzzi, Roberto Parisi, U.O. Semplice dipartimentale di Angiologia, Venezia; Caterina Brucoli, Giuseppe Palasciano, U.O. Medicina Interna Ospedaliera “Ferranini-Pende”, Bari; Giuseppe Camporese, Chiara Tonello, Azienda Ospedaliera di Padova, Padova; Lucia Canafoglia, Serena Rupoli, Clinica di Ematologia, Torrette di Ancona (ANCONA); Emilia Cancellieri, Oriana Paoletti, Sophie Testa, AO Istituti Ospitalieri di Cremona, Cremona; Anita Carlizza, Ospedale S. Giovanni dell’Addolorata, Roma; Marino Carnovali, Simona Sada, Anna Samaden, Azienda Ospedaliera “Guido Salvini”, Milano; Chiara Casarsa, Filippo Mearelli, Giulia Pivetti, S.C. Clinica Medica, Trieste; Roberto Catalini, Oriana Zingaretti, Medicina Vascolare, Torrette di Ancona (ANCONA); Stefania Cavazza, Benilde Cosmi, Angiologia e Malattie della Coagulazione “Marino Golinelli”, Bologna; Caterina Cenci, Domenico Prisco, Elena Silvestri, S.O.D. Patologia Medica, Firenze (FI); Fabrizio Ceresa, Francesco Patanè, Azienda Ospedaliera Ospedali Riuniti, Messina; Antonio Ciampa, Valeria Siniscalchi, Centro Emostasi, Avellino; Tiziana Ciarambino, Giuseppe De Bartolomeo, Ospedale Ferdinando Veneziale, Isernia; Michele Clemente, Presidio Ospedaliero “Madonna delle Grazie” - Matera, Matera; Franco Conti, Laura Paiella, A.O. San Camillo-Forlanini, Roma; Maria D’Avino, U.O.C. Medicina Interna ad indirizzo Angiologico e Cerebrovascolare, Napoli; Aldo D'Alessandro, Marisa Placentino, Vito Sollazzo, Ospedale Civile di San Severo (FG), San Severo (FG); Armando D'Angelo, Silvana Viganò, Servizio Coagulazione ed Unità Ricerca Trombosi Istituto Scientifico Universitario, Milano; Paolo De Campora, Raffaele Sangiuolo, Ospedale Buonconsiglio Fatebenefratelli, Napoli; Stefano De Franciscis, Raffaele Serra, Università degli Studi Magna Grecia di Catanzaro, Catanzaro; Egidio De Gaudenzi, SOC Medicina Interna, Domodossola (VB); Fernando De Santis, Giovanni Carlo Piccinni, Presidio Ospedaliero Francesco Ferrari-Casarano, Lecce; Italo De Tommaso, U.O. Cardiologia, A.O.R. San Carlo, Potenza; Letizia Di Francesco, Giovanni Maria Vincentelli, U.O.S. Reparto di Breve Osservazione, Roma; Rosario Di Maggio, Giorgia Saccullo, Sergio Siragusa, U.O. Ematologia con trapianto, Palermo; Pierpaolo Di Micco, Andrea Fontanella, Ospedale Buonconsiglio Fatebenefratelli, Napoli; Dario Di Michele, U.O. Medicina Interna - P.O.“G. Mazzini”, Teramo; Giovanni Di Minno, Antonella Tufano, Università Federico II di Napoli, Napoli; Marcello Di Nisio, Ettore Porreca, Dipartimento di Medicina e Scienze dell’invecchiamento, Chieti; Francesca Donadio, Davide Imberti, UOC di Medicina Interna ERI, Piacenza; Iolanda Enea, Azienda Ospedaliera S. Anna e S. Sebastiano di Caserta, Caserta; Fabio Fabbian, Roberto Manfredini, Marco Pala, Arcispedale Sant’Anna di Ferrara, Ferrara; Anna Falanga, Viola Milesi, Centro Di Emostasi e Trombosi, Bergamo; Valerio Fiore, Salvatore Santo Signorelli, Ospedale Garibaldi Centro – Catania, CATANIA (Catania); Elio Franco, Giorgio Giudice, U.O. Chirurgia Vascolare, Benevento; Gabriele Frausini, Marina Rovinelli, U.O.C. MEDICINA INTERNA, FANO (PU); Mariella Fuorlo, Raffaele Landolfi, Tiziana Morretti, U. O. Complessa Clinica Medica, Roma; Susanna Gamberini, Mauro Pasin, Raffaella Salmi, Arcispedale Sant’Anna di Ferrara, Ferrara; Angelo Ghirarduzzi, Maria Rosaria Veropalumbo, A.O. Arcispedale “Santa Maria Nuova”, Reggio Emilia; Mauro Ghizzi, Carlo Pepe, Azienda Unità Sanitaria Locale di Modena, Sassuolo Modena; Francesca Gianniello, Ida Martinelli, Fondazione IRCCS Ca’ Granda, MILANO; Diana Irina Iosub, Franco Piovella, FONDAZIONE IRCCS S. MATTEO DI PAVIA, PAVIA; Ercole Iozzi, Agostino Talerico, Unità Operativa di Angiologia, Crotone (KR); Micaela La Regina, Francesco Orlandini, Struttura Complessa Medicina Interna, La Spezia; Letizia Marconi, Antonio Palla, U.O. Pneumologia 1 Universitaria, Pisa; Rossella Marcucci, Daniela Poli, Dipartimento di Malattie Aterotrombotiche, Firenze (FI); Riccardo Margheriti, Gianpaolo Sala, OSPEDALE GRASSI, ROMA; Alfonso Marra, Cardiologia UTIC, Piedimonte Matese - Caserta; Fausto Marrocco, Ospedale Scolastica di Cassino, Cassino; Elisabetta Sara Montagna, Franco Silvestris, Simona Vallarelli, U.O. MEDICINA INTERNA UNIVERSITARIA “MICHELE BUFANO”, Bari; Lucio Mos, Valeria Rossetto, Dipartimento Di Emergenza, San Daniele del Friuli (UD); Francesco Mugno, Michelangelo Di Salvo, Presidio Ospedaliero Ferrarotto, Catania; Cinzia Nitti, Milena Pennacchioni, Aldo Salvi, Medicina Internistica e Sub-Intensiva, Torrette di Ancona (ANCONA); Oliviero Olivieri, Federica Tosi, Francesco Zorzi, Ospedale Borgo Roma, Verona; Maicol Onesta, U.O.C. di Medicina Interna, Fabriano (AN); Valeria Pagliara, Sabina Villalta, U.O. Medicina Interna 1, Treviso; Giancarlo Paolucci, Salvatore Severino, Casa di Cura Privata “Villa Serena”, CASSINO (FR); Francesca Pierri, Vitantonio Russo, U.O.C. Cardiologia, Taranto; Attilia Maria Pizzini, A.O. Arcispedale “Santa Maria Nuova”, Reggio Emilia; Roberto Quintavalla, Pasquale Rubino, Strutt. Complessa di Medicina interna ad indirizzo angiologico e coagulativo, Parma; Luigi Ria, Presidio Ospedaliero “Sacro Cuore di Gesù”, Lecce; Alessandro Schenone, S.C. Medicina Interna II, Genova; Cosimo Strafino, U.O. Medicina Interna, Termoli (CB); Pietro Tropeano, Centro Diagnosi e Cura Malattie Tromboemboliche Venose, Pordenone; Alfredo Vetrano, Azienda Ospedaliera S. Anna e S. Sebastiano di Caserta, Caserta; Nello Zanatta, U.O. Semplice Dipartimentale di Angiologia – Dipartimento di Medicina, Vittorio Veneto (TV).

### Spain

Maria Dolores Adarraga Cansino, Hospital de Montilla, Córdoba; Juan Alonso Gutierrez, Francisco Arnaiz de las Revillas, Hospital Valdecilla, Cantabria; Cristina Amado Fernández, Núria Calvo Mijares, Hospital de Sierrallana, Cantabria; Maria Ángeles Blanco-Molina, Hospital U. Reina Sofia Córdoba; Maria Angelina Garcia, Dolores Joya Seijo, Hospital Rey Juan Carlos, Móstoles; Rocío Aranda Blazquez, Juan-Bosco López-Sáez, Hospital U. Puerto Real, Puerto Real (Cádiz); Eduardo Arellano Rodrigo, Jaume Villalta Blanch, Hospital Clínic i Provincial, Barcelona; Arola Armengou Arxe, Fernando García-Bragado Dalmau, Hospital U. Dr. Josep Trueta, Girona; Aitor Ballaz Quincoces, Amaia García Loizaga, Hospital de Galdakao, Galdakao; José Luis Beato Pérez, Hospital de Hellín, Hellín (Albacete); Pedro Bedate Díaz, Andrés Quezada Loaiza, Hospital Central de Asturias, OVIEDO (PRINCIPADO DE ASTURIAS); Marc Cairols Castellote, Centro Médico Delfos, Barcelona; Inma Cañas Alcántara, Meritxell Lluís Padierna, Fundació Hospital de Granollers, Granollers; Marina Carrasco Expósito, Juan Antonio Millón Caño, Hospital de la Santa Creu i Sant Pau Barcelona; Amparo Carrasco Mas, Fernando Cereto Castro, Clínica Quirón, Barcelona; Rafael Castrodeza Sanz, Juan Ortiz de Saracho, Hospital El Bierzo, León; Elena Cisneros de la Fuente, Hospital Son Llàtzer, Palma de Mallorca; Cristina de Ancos Aracil, Justo Ruiz Ruiz, Hospital Universitario de Fuenlabrada, Madrid; Maria Dolores de Daborenea González, Alfonso Fernández Iglesias, Hospital U. de Cruces, Bizkaia; Javier de la Fuente Aguado, Lucia González González, Hospital POVISA, Vigo; Maria del Carmen Fernández-Capitán, Alicia Lorenzo Hernández, Hospital U. La Paz, Madrid; Jorge del Toro Cervera, Gloria Pérez Rus, Hospital G. U. Gregorio Marañón, Madrid; Juan Luis Delgado Bregel, Hospital Río Carrión, Palencia; Florentino Díez Fernández, Emilio-Antonio Santalla Valle, Complejo Asistencial Universitario de León, León; Teresa Elias Hernández, Luis Jara Palomares, Hospital Virgen del Rocío SEVILLA; Ramón Ferri Bataler, José Antonio Nieto Rodríguez, Hospital Virgen de la Luz, Cuenca; José María García García, Manuel Ángel Villanueva Montes, Hospital San Agustín, Áviles (Asturias); José Ramón González Porras, Hospital U. de Salamanca, Salamanca; María Guil García, Carlos Maria San Román Terán, Hospital Comarcal de la Axarquía, Málaga; Elena Hernando López, Alejandra Roncero Lázaro, Hospital San Pedro, La Rioja; María Jesús Jaras, Hospital de Cantoblanco, Madrid; David Jiménez Castro, Hospital Ramón y Cajal, Madrid; Rafael Jiménez-Rodríguez Madridejos, José María Pedrajas Navas, Hospital Clínico San Carlos, Madrid; Ramón Lecumberri, Nicolás Martínez, Clínica Universitaria de Navarra, Navarra; Genoveva Teresa López Castellanos, Luis Manzano Espinosa, Hospital Ramón y Cajal, Madrid; Luciano López Jiménez, Hospital U. Reina Sofia, Córdoba; Olga Madridano Cobo, Hospital Infanta Sofía, Madrid; Carmen Mainez Saiz, Yolanda Romero Pizarro, Hospital Puerta de Hierro, Madrid; Pablo Javier Marchena Yglesias, Parc Sanitari Sant Joan de Déu, Sant Boi de llobregat; Mar Martín del Pozo, Hospital Infanta Sofía, San Sebastián de los Reyes; Leonardo Melibovsky, Emilia Solé Altarriba, Hospital del Mar, Barcelona; Manuel Monreal Bosch, Hospital Germans Trias i Pujol, Badalona; Rafael Monte Secades, Hospital Universitario Lucus Augustí, Lugo; José Maria Mora Luján, Antoni Riera Mestre, Hospital Universitari de Bellvitge, Barcelona; Pedro Moral Moral, José Antonio Todolí Parra, Hospital U. La Fe, VALENCIA; Aurora Moreno Flores, Juan Francisco Sánchez Muñoz-Torrero, Hospital San Pedro de Alcántara, Cáceres; Francisco José Muñoz Rodríguez, Hospital de Mollet, Mollet del Vallès (Barcelona); Manuel J. Núñez Fernández, Complejo Hospitalario de Pontevedra, PONTEVEDRA; Estefanía Oncala Sibajas, Mercedes Vaquero de Sedas, Hospital Virgen de la Macarena, Sevilla; Pedro Parra Caballero, Hospital La Princesa, Madrid; Isaac Pons Martín del Campo, Hospital d'Igualada, Igualada; José Portillo Sánchez, Hospital G. U. Ciudad Real, Ciudad Real; Alberto Rivera Gallego, Iria Villaverde Álvarez, CHUVI - Hospital Xeral de Vigo, VIGO (Pontevedra); Eva Maria Rodríguez Beltrán, Demetrio Sánchez Fuentes, Complejo Hospitalario de Ávila, Ávila; Vanessa Roldán Schilling, Hospital Morales Messeguer, Murcia; Julio Sánchez Álvarez, Gregorio Tiberio López, Hospital Virgen del Camino, Pamplona; José Maria Suriñach Caralt, Hospital Universitario Vall d'Hebrón, Barcelona; Raimundo Tirado Miranda, Hospital Infanta Margarita, Cabra Córdoba; Esther Usandizaga de Antonio, Hospital Sant Joan Despí - Moisès Broggi, St. Joan Despí.

### Switzerland

Martin Banyai, Luzerner Kantonspital, Luzern; Ulrich Frank, Gian Reto Jörg, Kantonspital Graubünden, Chur; Christina Jeanneret, Kantonsspital Bruderholz, Bruderholz; Daniel Staub, Universitätsspital Basel, Basel.

### United Kingdom

Sam Ackroyd, Bradford Royal Infirmary, Bradford; Gaurav Agarwal, Ben Mearns, East Surrey Hospital, Redhill; Raza Alikhan, University Hospital Wales, Cardiff; Allameddine Allameddine, The Royal Oldham Hospital, Manchester; Faris Al-Refaie, The Princess Alexandra Hospital NHS Trust, Essex; Christopher Arden, Park Surgery, Chandlers Ford; Antony Austin, The Meadows Surgery, Ilminster; Ameet Bakhai, Barnet Hospital, Barnet; Thomas Barton, Hayder Ewad, Neath Port Talbot Hospital, Port Talbot; Richard Body, Jecko Thachil, Manchester Royal Infirmary, Manchester; Joseph Chacko, Royal Bournemouth Hospital, Bournemouth; Deepak Chandra, University Hospital of North Staffordshire NHS Trust, Stoke-on-Trent; Freda Charters, Burnfield Medical Practice, Inverness; Alistair Church, Frances McGrane, Western Infirmary, Glasgow; John Clements, The Elmwood Surgery, Belfast; Piers Clifford, Wycombe Hospital, Wycombe; Dominic Cox, Northampton General Hospital, Northampton; Matthew Crouch, The Practice of Health, Barry; Mark Crowther, Worcestershire Royal Hospital, Worcester; Emyr Davies, Waterfront Medical Centre, Barry; Mark Davies, West Cross Medical Centre, Swansea; Sameh Dimitri, Countess of Chester Hospital, Chester; Anja Drebes, Royal Free Hospital, London; Simon Franklin, Budleigh Salterton Medical Centre, Devon; Jacob George, Nicola Irvine, Ninewells Hospital, Dundee; Hagen Gerofke, Basildon University Hospital, Essex; Christopher Gibbs, North Devon District Hospital, Barnstaple; Teik Goh, The Garth Surgery, Cleveland; Sunil Gupta, Eastbourne District General and Conquest Hospital, Eastbourne; Jon Holmes, Bronglais General Hospital, Aberystwith; Ewart Jackson-Voyzey, Axbridge & Wedmore Research Practice, Axbridge; Nick Jones, St Chad's Surgery, Radstock; Arun Kallat, Royal Bolton Hospital, Bolton; Patrick Kerr, The Wall House Surgery, Surrey; Patrick Kesteven, Newcastle upon Tyne Hospital Trust, Newcastle upon Tyne; Tristan Lench, Severnbank Surgery, Lydney; William Lester, Gillian Lowe, University Hospital Birmingham, Birmingham; Martin Lewis, The Queen Elizabeth Hospital NHS Foundation Trust, Norfolk; Terry McCormack, Whitby Group Practice, Whitby; Andrew McCoye, Ecclesfield Group Practice, Sheffield; Andrew Moriarty, Craigavon Area Hospital, Belfast; Wendy Morris, The Birches, Solihull; Bethan Myers, Lincoln County, Lincoln; Mekkali Narayanan, George Eliot Hospital NHS Trust, Nuneaton; New Oo, Huddersfield Royal Infirmary, Huddersfield; Matthew Reed, Royal Infirmary of Edinburgh, Edinburgh; Peter Rose, Warwick Hospital, Warwick; Khalid Saja, Queen's Hospital, Romford; Muttuswamy Sivakumaran, Peterborough City Hospital, Peterborough; Mamta Sohal, Ealing Hospital, Southall; Gary Solomons, Parkwood Surgery, Hemel Hempstead; Sayed J. Sultanzadeh, Doncaster Royal Infirmary, Doncaster; Tamsin Venton, Teign Estuary Medical Group, Glendevon Medical Centre, Teignmouth; John Wakeling, Ely Bridge Surgery, Cardiff; Ceri Walby, Clifton Surgery, Cardiff; Michael Waldron, Enchord Limited, Cornwall; Simon Watt, Wythenshawe hospital, Manchester; William Willcock, Rolle Medical Partnership, Exmouth; Azhar Zafar, Danes Camp Medical Centre, Northampton.

## Abbreviations

VTE: Venous thromboembolism
PE: Pulmonary embolism
DVT: Deep vein thrombosis
UFH: Unfractionated heparin
LMWH: Low molecular weight heparin
VKA: Vitamin K antagonist
INR: International normalization ratio
QoL: Quality of life
HR-QoL: Health related quality of life
CV: Cardiovascular
CRO: Contact research organization
PACT-Q2: Perception of anticoagulant treatment questionnaire 2
Veines-QoL/Sym: Venous Insufficiency Epidemiological and Economic Study
PEmb-QoL: Pulmonary Embolism Quality of Life
AE: Adverse event
CI: Confidence interval
